# Identification of *CqCYP76AD5v1*, a gene involved in betaxanthin biosynthesis in *Chenopodium quinoa*, and its product, betaxanthin, which inhibits amyloid-β aggregation

**DOI:** 10.5511/plantbiotechnology.25.0122a

**Published:** 2025-06-25

**Authors:** Tomohiro Imamura, Hironori Koga, Akio Miyazato, Zhe Xu, Ryouta Shigehisa, Shinya Ohki, Masashi Mori

**Affiliations:** 1 Department of Bioproduction Science, Ishikawa Prefectural University; 2 Center for Nano Materials and Technology (CNMT), Japan Advanced Institute of Science and Technology (JAIST); 3 Research Institute for Bioresources and Biotechnology, Ishikawa Prefectural University

**Keywords:** amyloid-β, betalain, betaxanthin, *CqCYP76AD5v1*, quinoa

## Abstract

Betalain pigments, primarily produced by the order Caryophyllales, are categorized into betacyanins (red/purple) and betaxanthins (yellow/orange). While the biosynthetic pathways of these pigments are well-studied, the genes responsible for betaxanthin biosynthesis in quinoa were previously unknown. This study identified three candidate genes, *CqCYP76AD5v1*, *CqCYP76AD5v2*, and *CqCYP76AD130*, as quinoa orthologs of beet *CYP76AD5* and *CYP76AD6*. Agroinfiltration experiments in *Nicotiana benthamiana* revealed that *CqCYP76AD5v1* exhibited L-DOPA synthesis activity, whereas *CqCYP76AD130* did not. To enable large-scale production of betaxanthins, we developed a tobacco BY-2 cell line expressing *CqCYP76AD5v1* and *CqDODA1-1*, with vulgaxanthin I identified as the predominant product. Furthermore, the betaxanthin mixture extracted from this line inhibited amyloid-β (Aβ) aggregation, a key factor associated with Alzheimer’s disease. These findings demonstrate the potential of betaxanthins derived from quinoa betaxanthin-biosynthesis genes for applications in health supplements and pharmaceuticals.

## Introduction

Betalains are natural pigments produced by Caryophyllales plants (Brockington et al. 2011) and some basidiomycetes (Gill 1994). Plants that produce betalains do not produce anthocyanins (Tanaka et al. 2008). In contrast to anthocyanins, betalains retain nitrogen atoms within their molecular structure and demonstrate high antioxidant capacity (Wybraniec et al. 2011). Betalains accumulate in various tissues, including flowers, storage tissues, stems, and leaves. Their production is induced by light and environmental stresses (Hinojosa et al. 2018). Betalains protect plants against abiotic and nonbiotic stresses (Jain et al. 2015; Polturak et al. 2017). Moreover, betalains exhibit a range of biological activities (bioactivities), including anticancer, anti-inflammatory, and anti-bacterial activities (Madadi et al. 2020). Previously, our group has demonstrated that betacyanins possess inhibitory properties against HIV-1 protease and amyloid-β (Aβ) aggregation (Imamura et al. 2019, 2022), however, in betaxanthin, their biological effects are unknown. In light of these findings, betalains, which are popular for their vibrant hues, are expected to be incorporated into medicinal products and dietary supplements, given their bioactivity and use as a coloring agent.

**Figure 1:**
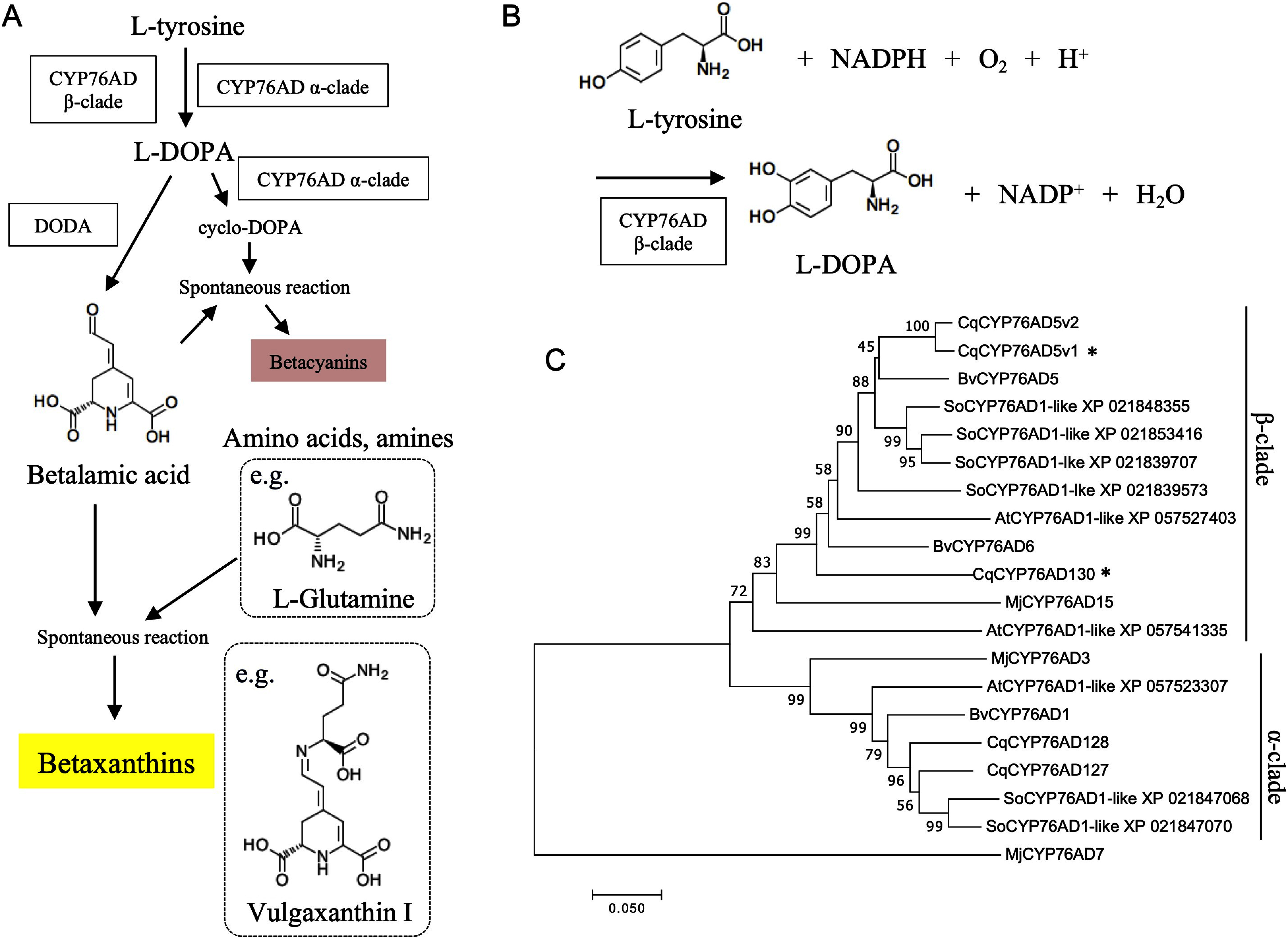
Figure 1. Betaxanthin biosynthetic genes. (A) Scheme for the betaxanthin biosynthetic pathway. Boxes indicate the betaxanthin biosynthetic enzyme. CYP76AD β-clade, cytochrome P450 76AD β-clade; CYP76AD α-clade, cytochrome P450 76AD α-clade; DODA, DOPA 4,5-dioxygenase. The chemical structures of vulgaxanthin I and L-glutamine, a component of vulgaxanthin I, are shown as examples. (B) Schematic representation of the reaction of L-DOPA involving CYP76AD β-clade. (C) Molecular phylogenetic tree of the α- and β-clade CYP76AD family based on amino acid sequences. Multiple sequences were aligned using ClustalW and used for tree construction utilizing the maximum likelihood method of MEGA11. Bootstrap values from 5000 replicates are shown on branches. The bar represents 0.050 amino acid substitutions per site. Details of the flavonoid glycosyltransferase homologs from other plant species are provided in Supplementary Table S1. Asterisks indicate genes cloned in this study.

In plants that produce betalains, such as four-o’clock, beet, and quinoa, the betalain biosynthetic pathway begins with the hydroxylation of L-tyrosine to form L-3,4-dihydroxyphenylalanine (L-DOPA) catalyzed by the redundant cytochrome P450 enzymes belonging to CYP76AD α-clade, and CYP76AD β-clade (Figure 1A, B) (Polturak et al. 2016; Sunnadeniya et al. 2016). L-DOPA is then converted to betalamic acid by DOPA 4,5-dioxygenase (Christinet et al. 2004; Gandía-Herrero and García-Carmona 2012) or cyclo-DOPA by CYP76AD α-clade (Hatlestad et al. 2012). In betaxanthin biosynthesis, betalamic acid can spontaneously condense with amino acids or other amine-containing compounds to form yellow fluorescent betaxanthins (Figure 1A) (Schliemann et al. 1999). In betacyanin biosynthesis, betanidin, the backbone of betacyanin, is synthesized by the spontaneous condensation of betalamic acid and cyclo-DOPA (Steiner et al. 1999). Betalain-related glucosyltransferases that catalyze the 5-*O*-glucosylation of cyclo-DOPA (Sasaki et al. 2005) or alternatively the 5-*O*- or 6-*O*-glucosylation of betanidin (Das et al. 2013; Vogt 2002; Vogt et al. 1999) can further modify betacyanins. Furthermore, our group had isolated a gene that synthesizes amaranthin by binding glucuronic acid to betanin (Imamura et al. 2019).

Quinoa (*Chenopodium quinoa*) is a pseudo-cereal grain belonging to Amaranthaceae, indigenous to the Andes. It is remarkably resilient to environmental stresses, including salt and drought, and can thrive in conditions unsuitable for other crops (Hinojosa et al. 2018). Furthermore, quinoa grains possess high nutritional value (Vega-Gálvez et al. 2010). Therefore, quinoa may help alleviate food insecurity (Bazile et al. 2015). Regarding quinoa research, the quinoa genome sequence has already been determined by multiple research groups (Jarvis et al. 2017; Yasui et al. 2016). The elucidation of the genome sequence has facilitated molecular genetics and molecular biological studies. Consequently, genes involved in the development of bladder cells, the distinctive epidermal cells of quinoa, were identified (Imamura et al. 2020). Quinoa can also produce betalains, primarily betacyanins (red betalains). These include betanin, amaranthin, and celosianin II, which are produced and accumulated (Imamura et al. 2018). To date, our group has succeeded in isolating a cluster of betacyanin biosynthesis genes in quinoa (Imamura et al. 2018, 2019). In quinoa varieties with yellow-colored seeds, the primary yellowing factor is betaxanthin (yellow betalains), including the accumulation of dopaxanthin, miraxanthin V, and indicaxanthin (Escribano et al. 2017). A recent study reported that 6-decarboxy-betaxanthin is produced from dopamine via 6-decarboxy-betalamic acid (Henarejos-Escudero et al. 2021). The characterization of the betaxanthin biosynthesis pathway in quinoa has led to the identification of *DODA*, which functions in common with betacyanins (Imamura et al. 2018). However, the β-clade gene for the other *CYP76AD* gene remains unknown.

In this study, we attempted to isolate *CYP76AD* and analyze its function to identify the genes involved in betaxanthin biosynthesis in quinoa. A homology search using the amino acid sequences of beet *CYP76AD5* and *CYP76AD6* resulted in three orthologs: *CqCYP76AD5v1, CqCYP76AD5v2*, and *CqCYP76AD130*. We attempted to isolate genes from quinoa seedlings and successfully cloned *CqCYP76AD5v1* and *CqCYP76AD130*. However, *CqCYP76AD5v2* could not be cloned due to the lack of detectable expression. The capacity for betaxanthin synthesis (L-DOPA synthesis, Figure 1B) was evaluated through agroinfiltration for *CqCYP76AD5v1* and *CqCYP76AD130*. The results demonstrated that *CqCYP76AD5v1* possesses L-DOPA synthesis activity. An artificial betaxanthin production system was established using the refined mass production system utilizing the isolated *CqCYP76AD5v1*. Furthermore, betaxanthins were employed to identify new bioactivities. Thus, the isolation of the betaxanthin biosynthetic gene will facilitate the discovery of new bioactivities and development of artificial systems for industrial betacyanin production.

## Materials and methods

### Plant materials and growth conditions

Seeds of the quinoa variety CQ127 were obtained from the U.S. Department of Agriculture. Seeds were sown in a cell tray and grown at 23°C under a 12-h light/12-h dark photoperiod in a phytotron. Two-week old seedlings were transplanted in to 5-L plant pots with standard potting mix (Ikubyou Baido, Takii, Kyoto, Japan) and grown in a glasshouse. Tobacco BY-2 cells were grown in Linsmaier and Skoog medium supplemented with 3% sucrose and 0.2 mg l^−1^ 2,4-dichlorophenoxyacetic acid at 26°C (Nagata et al. 1992).

### Phylogenetic tree of deduced amino acid sequences

The ClustalW algorithm was used to align the deduced amino acid sequences of the β-clade genes of the *CYP76AD* family from other plant species (Supplementary Table S1). The neighbor-joining algorithm of the MEGA11 software was used to construct a phylogenetic tree (Tamura et al. 2021).

### Molecular cloning

To eliminate genomic DNA, total RNA was extracted from quinoa hypocotyl using an RNeasy Plant Mini kit (Qiagen, Valencia, CA, USA) and treated with RNase-free DNase I (Nippon Gene, Tokyo, Japan). A TaKaRa RNA PCR kit (AMV) ver. 3.0 (TaKaRa, Kusatsu, Japan) with oligo(dT) primers was used to synthesize first-strand cDNA from 500 ng total RNA. The full-length ORF sequences of *CqCYP76AD5v1* (XM_021920495) and *CqCYP76AD130* (XM_021861500) were obtained using gene specific primers (Supplementary Table S2).

### Plasmid construction

PCR amplification was performed using PrimeSTAR GXL DNA polymerase and oligonucleotides containing a restriction enzyme cleavage site (Supplementary Table S2). The amplified fragments of *CqCYP76AD5v1* and *CqCYP76AD130* were digested with the appropriate restriction enzymes and then introduced into the binary vector pCAMBIA1301MdNcoI (Imamura et al. 2018). pCAM-CqDODA-1 was constructed previously (Imamura et al. 2018). Plasmid sequencing was performed using BigDye terminator chemistry and an ABI PRISM 3100 genetic analyzer (Applied Biosystems, Foster City, CA, USA).

### Transient expression in *N. benthamiana*

Using the triparental mating method, the expression constructs were transformed into *Agrobacterium tumefaciens* strain GV3101 (Wise et al. 2006). The agroinfiltration method followed the procedure described previously (Imamura et al. 2018). Briefly, each transformed *Agrobacterium* was cultured overnight at 25°C, 130 rpm in 3 ml of LB medium. The cultured *Agrobacterium* strains included those harboring the genes to be assessed (*CqCYP76AD5v1*, *CqCYP76AD130*, and *AcGFP1* as a control), along with *Agrobacterium* harboring *CqDODA-1* and *P19*, an RNA silencing suppressor (Silhavy et al. 2002). These strains were mixed, and the optical density at 600 nm (OD600) was adjusted to 0.1 using sterile water for agroinfiltration. The adjusted *Agrobacterium* mixture was then infiltrated into the leaves of 5- to 6-week-old *N. benthamiana* plants using a syringe. The infiltrated plants were cultivated in a growth chamber at 23°C and 60% humidity under long day conditions (16-h light/8-h dark cycle). Subsequent analyses were performed on the infiltrated leaves 5 days after infiltration.

### BY-2 cells transformation

Tobacco BY-2 cells were grown in a Linsmaier and Skoog medium supplemented with 3% sucrose and 0.2 mg l^−1^ 2,4-dichlorophenoxyacetic acid at 26°C (Nagata et al. 1992). The *A. tumefaciens* strain GV3101, which harbors a Ti plasmid, was used to transform the cells as described previously (Hagiwara et al. 2003). Transgenic lines were selected on an agar medium containing the appropriate selective agents, namely, 50 mg l^−1^ hygromycin with 500 mg l^−1^ carbenicillin. During primary screening, suspension cells derived from calli were grown in 3 ml of liquid medium in six-well culture plates and then transferred to 150 ml of liquid medium in 500-ml flasks while shaken constantly at 135 rpm. After the initial culture for 2–3 weeks, the suspension cells were maintained without selective agents.

### RT-PCR analysis

A High Capacity cDNA Reverse Transcription kit (Thermo Fisher Scientific, Waltham, MA, USA) with random primers was used to synthesize first-strand cDNA from 500 ng of total RNA. A GeneAtlas 322 (Astec, Fukuoka, Japan) with PrimeSTAR GXL DNA Polymerase (TaKaRa) was used to perform RT-PCR. The procedure for amplification of the candidate transcripts comprised initial denaturation at 94°C for 2 min, followed by 35 cycles at 98°C for 10 s, 55°C for 15 s, and 68°C for 1.5 min. *L23* and *NtCesA* were used as positive controls for the expression in *N. benthamiana* leaves and tobacco BY-2 cells, respectively (Grimberg et al. 2015; Nakagawa and Sakurai 2001). Primer pairs are listed in Supplementary Table S2.

### Plant pigment analysis

Pigments extracted from tobacco BY-2 cells were analyzed as described previously (Imamura et al. 2019), and extracts were concentrated using a centrifugal concentrator (CC-105, Tomy Seiko Inc., Tokyo, Japan). A Shimadzu LC-20AD system (Kyoto, Japan) was used for analytical high-performance liquid chromatography (HPLC) separations. Samples were separated on a Shim-pack GWS C18 column (5 µm; 200×4.6 mm i.d.; Shimadzu GLC). Linear gradients were run from 0% B to 45% B over 45 min using 0.05% trifluoroacetic acid (TFA) in water (solvent A) and 0.05% TFA in acetonitrile (solvent B) at a flow rate of 0.5 ml min^−1^ at 25°C. The elution was monitored by absorbance at 536 nm.

### Liquid chromatography-mass spectrometry (LC-MS) analysis

The purified quinoa-derived betacyanins was confirmed by LC-MS performed using a Shimadzu LC-20AD system equipped with an electrospray ionization Fourier transform ion cyclotron resonance mass spectrometer (Solarix, Bruker Daltonics, Billerica, MA, USA) in the positive mode. For separation, an XBridge C18 column (150×2.1 mm) with a 3.5-µm particle size (Waters, Framingham, MA, USA) was used. Using 0.1% TFA in acetonitrile, the flow rate was 0.3 ml min^−1^. A stepwise gradient using 0, 10, 50, and 100% acetonitrile at 0–3, 3–15, 15–20, and 20–25 min, respectively, was employed.

### Betaxanthin sample preparation

An aqueous solution of betaxanthins from the transgenic BY-2 line and non-transgenic line were purified using anion-exchange chromatography (DEAE Sepharose Fast Flow, Cytiva, Uppsala, Sweden) and reversed-phase chromatography (COSMOSIL 75C_18_-OPN, Nacalai Tesque, Kyoto, Japan) as described by Henarejos-Escudero et al. (2018). The absorbance spectrum of betaxanthins from the transgenic BY-2 line was measured in the range of 300 nm to 800 nm. The measurements were performed using a UV-2450 spectrophotometer (Shimadzu). Betaxanthin concentration was determined using the molar extinction coefficient of ε=48,000 M^−1^ cm^−1^ at 480 nm (Henarejos-Escudero et al. 2018) through UV-Vis spectroscopy (Shimadzu). The eluate was evaporated to dryness, and the residues were dissolved in water and stored at −20°C until use.

### Thioflavin T (ThT) fluorescence assay

The ThT fluorescence assay was performed using the SensoLyte Thioflavin T β-Amyloid Aggregation Kit (ANASPEC, Fremont, CA, USA) as described previously (Imamura et al. 2022). Aβ40 and ThT solutions of (50 µM and 200 µM, respectively) were used in the evaluation system, according to the manufacturer’s protocol. Aβ40 was dissolved in phosphate-buffered saline solution (PBS, pH 7.4). In this study, a 50 µM betaxanthin mixture and extracts from non-transformed plants (NT) were used as samples for evaluation. Water was used as a positive control. ThT fluorescence was measured at 37°C using a spectrofluorometer (Varioskan LUX; Thermo Fisher Scientific) at Ex/Em=440/484 nm. Readings were taken every 5 min, and immediately before each reading, each sample was shaken for 15 s. Fluorescence data were analyzed using Skanlt software (Thermo Fisher Scientific). The values presented are an average of four wells.

### Transmission electron microscopy (TEM) of Aβ aggregates

TEM was conducted based on previous studies (Imamura et al. 2022). Aβ40 was solubilized in DMSO to give a 1 mM solution. Aβ solutions were prepared with and without adding 50 µM betaxanthin mixture or a non-transgenic extract and diluted to 20 µM with PBS. Before TEM observations, Aβ mixtures were incubated at 37°C for 5 days. Approximately 2 µl of the sample solution was placed on a 150-mesh copper grid covered with formvar. After 5 min, the sample was soaked away, and the grid was stained with 2.0% (w/v) uranyl acetate solution. TEM images of Aβ aggregates were obtained using a Hitachi 7650 transmission electron microscope (Hitachi Co., Ltd., Tokyo, Japan) with an acceleration voltage of 80 kV.

## Results

### Search for the β-clade of *CYP76AD* gene in the quinoa genome

Betaxanthin biosynthesis requires the β-clade gene of *CYP76AD*. A homology search using the amino acid sequence of BvCYP76AD5 and BvCYP76AD6 in beet by NCBI BLASTp. It revealed that two orthologs of *BvCYP76AD5* in quinoa: *CqCYP76AD5v1*, *CqCYP76AD5v2*, and that one orthologs of *BvCYP76AD6* in quinoa: *CqCYP76AD130*. The identity rates of the quinoa orthologs to BvCYP76AD5 were 87.6% and 88.2% for CqCYP76AD5v1 and CqCYP76AD5v2, respectively. The identity rates of the quinoa orthologs to BvCYP76AD6 was 82% for CqCYP76AD130. In the phylogenetic tree analysis using the β-clade of CYP76ADs from other plant species, including beet, all CqCYP76ADs belonged to the β-clade CYP76ADs (Figure 1C).

### Identification of *CqCYP76AD* β-clade genes

**Figure 2:**
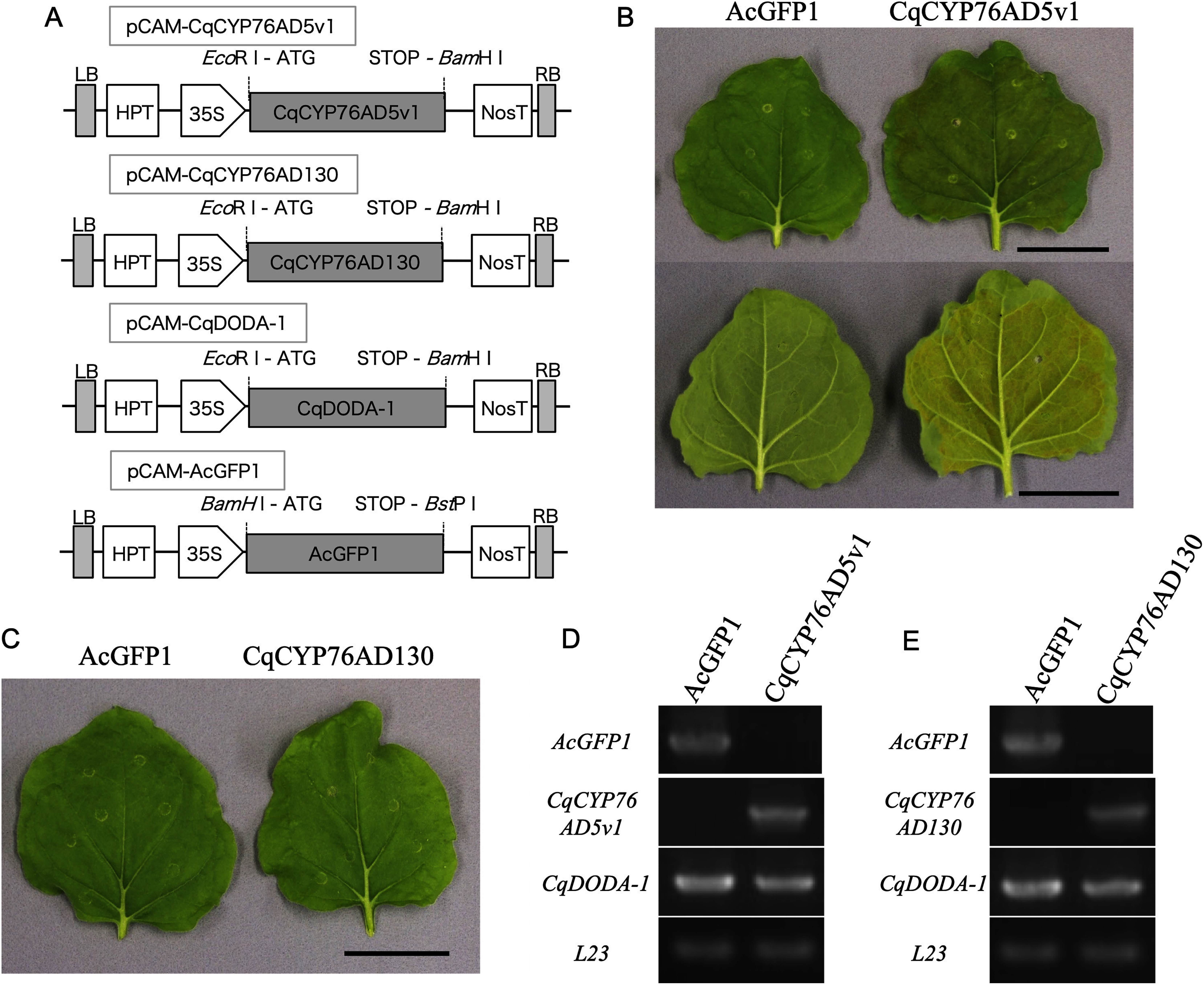
Figure 2. Identification of β-clade genes of the *CYP76AD* family in quinoa. (A) Schematic representations of the plant expression vectors. CqCYP76AD5v1, *CqCYP76AD5v1* CDS; CqCYP76AD130, *CqCYP76AD130* CDS; CqDODA-1, *CqDODA-1* CDS; AcGFP1, *AcGFP1* CDS; 35S, CaMV 35S promoter; 35S-T, 35S terminator; ATG, start codon; HPT, *hygromycin phosphotransferase* expression cassette; LB, left border; NosT, *nopaline synthase* terminator; RB, right border; STOP, stop codon. (B) Recombinant expression of *CqCYP76AD5v1* in *N. benthamiana* leaves. CqCYP76AD5v1 indicates co-infiltration of transgenic *Agrobacterium* harboring plasmids containing *CqCYP76AD5v1*, *CqDODA-1*, and *P19*. The upper and lower panels represent the adaxial and abaxial sides of the infected leaf of *N. benthamiana*, respectively. (C) Recombinant expression of *CqCYP76AD130* in *N. benthamiana* leaves. CqCYP76AD130 shows co-infiltration of transgenic *Agrobacterium* harboring plasmids containing *CqCYP76AD130*, *CqDODA-1*, and *P19*. AcGFP1 means co-infiltration of transgenic *Agrobacterium* harboring plasmids containing *AcGFP1*, *CqDODA-1*, and *P19* as a negative control. Bars=4 cm. (D, E) RT-PCR analysis of the gene expression in infiltrated leaves of *N. benthamiana*. (D) CqCYP76AD5v1 indicates co-expression with *CqCYP76AD5v1* and *CqDODA-1* in *N. benthamiana* leaves. (E) CqCYP76AD130 indicates co-expression with *CqCYP76AD130* and *CqDODA-1* in *N. benthamiana* leaves. *AcGFP1* indicates control. *L23* indicates an internal control.

To analyze the β-clade genes of the quinoa *CYP76AD* family, genes were isolated from the cDNA of 2-week-old quinoa seedlings. As a result, *CqCYP76AD5v1* and *CqCYP76AD130* were successfully cloned. However, the expression of *CqCYP76AD5v2* could not be detected, and it was therefore not cloned. The lengths of the ORFs of *CqCYP76AD5v1* and *CqCYP76AD130* were 1,515 bp and 1,500 bp, respectively. The L-DOPA synthesis activity of the successfully cloned *CqCYP76AD5v1* and *CqCYP76AD130* was subsequently evaluated. Plant expression plasmids (Figure 2A) were constructed for these genes and introduced into the *Agrobacterium* strain. *Agrobacterium* carrying a gene (*CqCYP76AD5v1*, *CqCYP76AD130* or *AcGFP1* as a negative control) to assess betaxanthin production, an RNA silencing repressor (*P19*; Silhavy et al. 2002), and another *Agrobacterium* carrying the betaxanthin biosynthesis gene *CqDODA-1* were mixed together. The resulting transformed *Agrobacterium* mixture was injected into the leaves of *N. benthamiana* tobacco using the agroinfiltration method, and its L-DOPA synthesis activity was evaluated. Agroinfiltration results revealed betaxanthin-derived yellow coloration in *CqCYP76AD5v1*-infected leaves (Figure 2B). In contrast, no coloration was observed in *CqCYP76AD130*-infected leaves (Figure 2C). The expression of those transgenes in these infiltrated leaves was assessed using RT-PCR, confirming that all the transgenes were expressed (Figure 2D, E). The results showed L-DOPA synthesis activity for CqCYP76AD5v1. In contrast, CqCYP76AD130 failed to exhibit L-DOPA-synthesizing activity. The amino acid sequences of the two proteins were analyzed for differences in their L-DOPA biosynthetic activity. A mutation (D301N) was identified in CqCYP76AD130 at an amino acid residue within the chemical substrate-binding pocket, which is conserved across the CYP76 family (Supplementary Figure S1).

### Production of betaxanthin pigments in BY-2 cells

**Figure 3:**
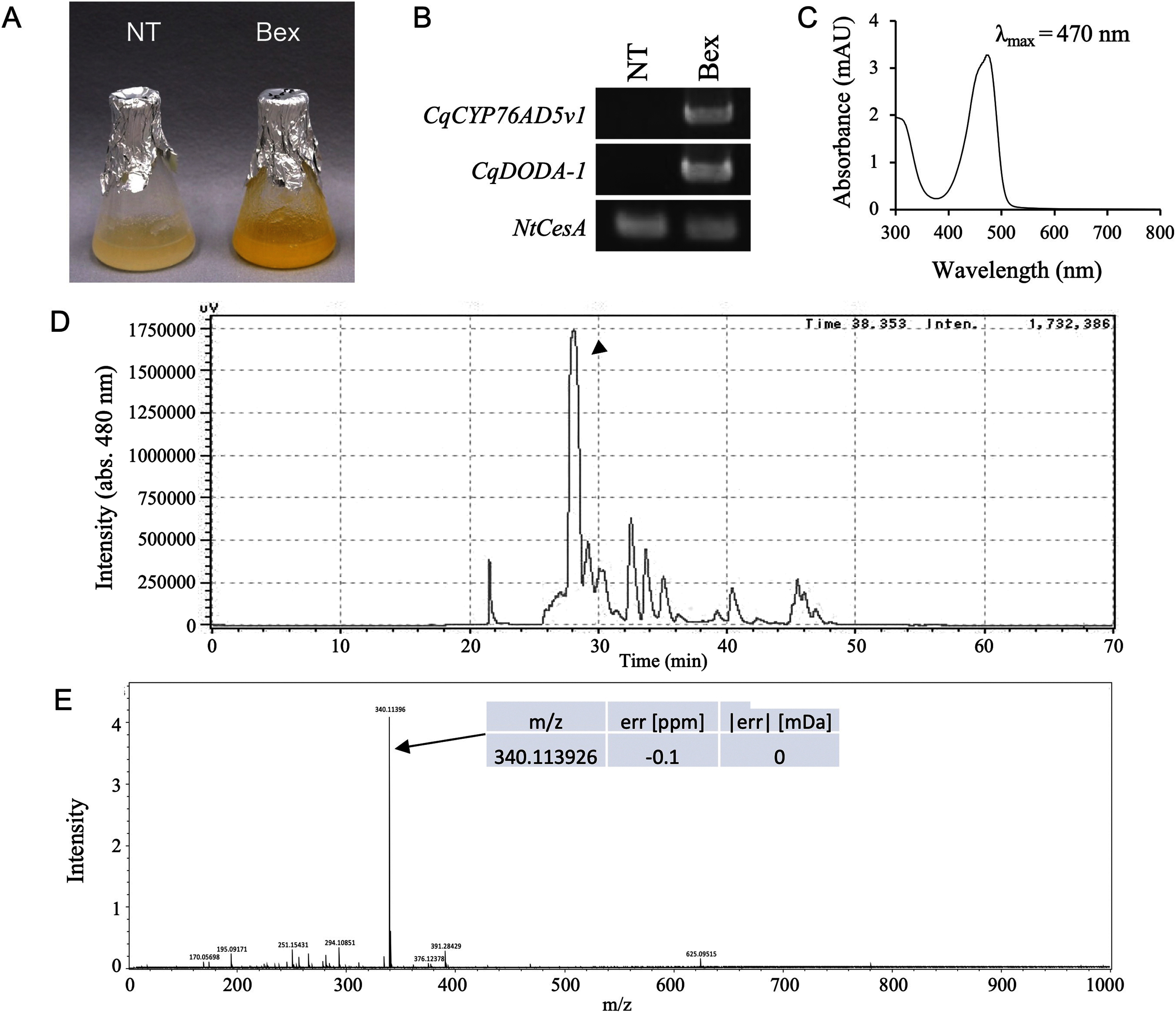
Figure 3. Production of betaxanthin pigments in BY-2 cells. (A) Photographs of the transformed BY-2 cell lines 2 weeks after transplantation. Bex indicates the transgenic BY-2 cell line producing betaxanthins. NT denotes the non-transgenic BY-2 cell line. (B) RT-PCR analysis of gene expression in transformed BY-2 cells. Bex indicates co-expression with *CqCYP76AD5v1* and *CqDODA-1* in tobacco BY-2 cells. *NtCesA* indicates an internal control. (C) UV-vis spectra of betaxanthin mixture produced by Bex line. The horizontal and vertical axes indicate absorbance (mAU) and wavelength (nm), respectively. (D) HPLC chromatograms of extracts of the Bex line. The arrowhead indicates the elution fraction that was analyzed by mass spectrometry. The horizontal and vertical axes indicate the retention time (min) and signal intensity (µV), respectively. (E) MS spectra of HPLC elution samples from the Bex line extract. The upper and lower panels indicate the HPLC elution samples at 28 min (arrowhead in D). The HPLC elution samples at 28 min indicate vulgaxanthin I (arrow). The horizontal and vertical axes indicate the mass-to-charge ratio (*m*/*z*) and relative abundance, respectively.

*CqCYP76AD5v1* and *CqDODA-1* are required for betaxanthin synthesis in quinoa. Therefore, these genes were introduced into BY-2 cells, and mass production of betaxanthins was attempted. The transformed *Agrobacterium* carrying the expression plasmids used for agroinfiltration were produced and introduced into BY-2 cells. A betaxanthin-producing cell line harboring *CqCYP76AD5v1* and *CqDODA-1* was established (Figure 3A). After confirming transgene expression by RT-PCR (Figure 3B), a cell line exhibiting intense coloration was selected and grown in a liquid culture. The betaxanthin-producing cell line (Bex line) was vivid yellow (Figure 3A). The absorbance spectrum of the dye extracted and purified from this cell line was measured using UV-Vis spectroscopy, with a λ_max_ of 470 nm (Figure 3C). This value is within the same region as the λ_max_ of betaxanthin. Several betaxanthin-derived peaks were observed in the Bex line (Figure 3D). Among these peaks, a significant accumulation of material was detected at an HPLC elution time of 28 min (Figure 3D). Mass spectrometry analysis of this 28-min elution peak revealed the same *m*/*z* value as that of vulgaxanthin I, a glutamine-derived betaxanthin (Figures 1A, 3E). Polturak et al. (2017) reported that the λ_max_ of vulgaxanthin I is 470 nm and that vulgaxanthin I production was increased in BY-2 cells. This suggests that the betaxanthin produced in the Bex line is primarily vulgaxanthin I. Therefore, a betaxanthin mixture was successfully produced artificially using the betaxanthin biosynthesis gene from quinoa. The Bex line produced approximately 7.1±1.6 µM of betaxanthin per liter of BY-2 cells over a two-week period.

### Betaxanthin mixture inhibits Aβ aggregation

**Figure 4:**
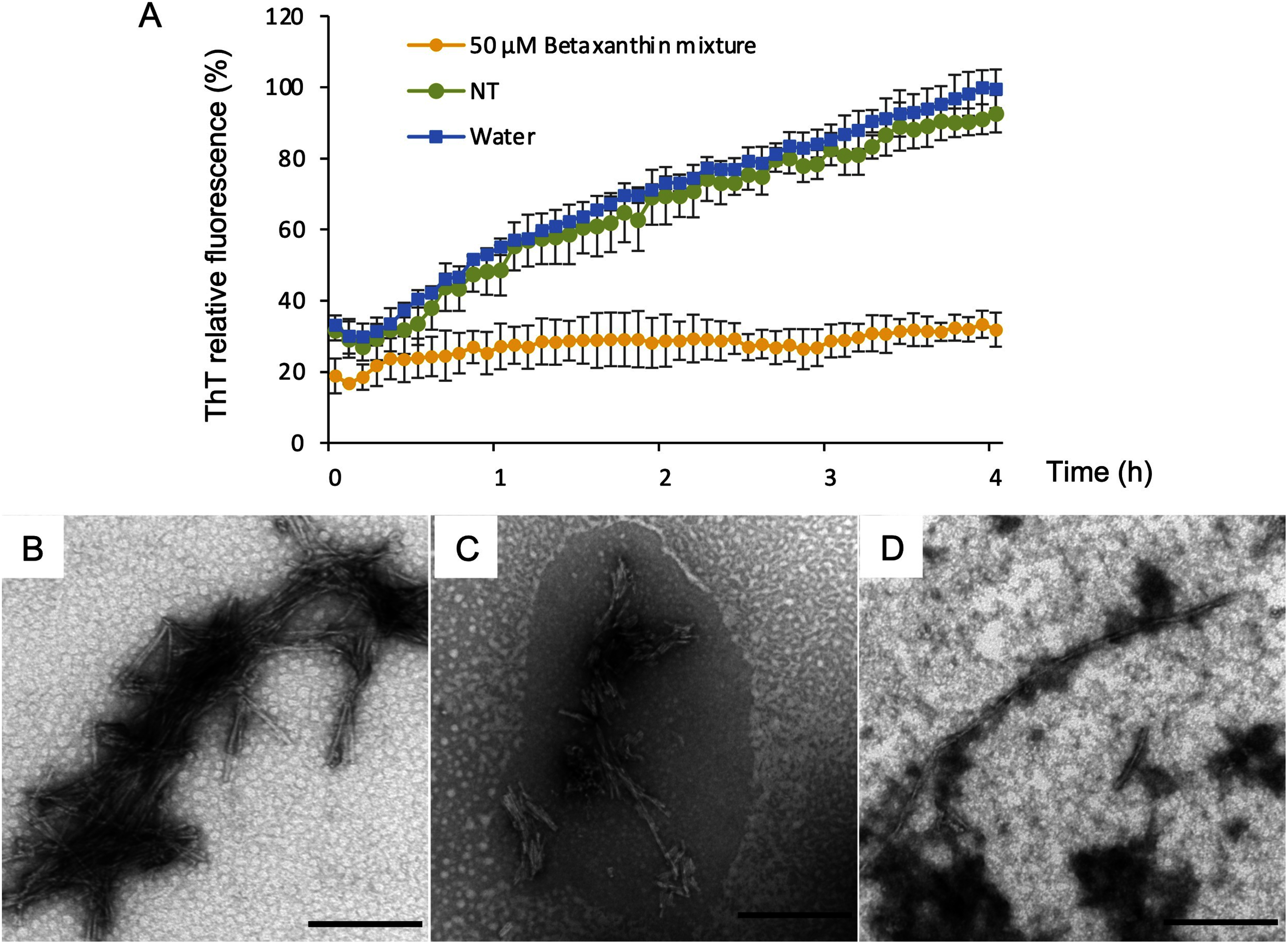
Figure 4. Evaluation of inhibitory activity against human amyloid-β 40 (Aβ40) aggregation. (A) Thioflavin T (ThT) fluorescence readings for Aβ40 incubated with betaxanthin. Aβ40 was incubated with a 50 µM betaxanthin mixture, an extract from non-transgenic BY-2 cells (NT), and water. NT is an extract from non-transgenic BY-2 cells grown in 100 ml cultures, processed using the same extraction and purification methods as those for betaxanthin. The error bars represent the means±SDs (*n*=4). The ThT relative fluorescence was expressed as a percentage of the fluorescence of water, with a maximum value of 100%. (B–D) Transmission electron microscope images of Aβ40 aggregates. (B) Aβ40 alone. (C) Aβ40 with extract of non-transgenic BY-2 cell. (D) Aβ40 with 50 µM betaxanthin mixture. Scale bars=200 nm.

To uncover new bioactivities of betaxanthin, the ThT fluorescence assay was performed to evaluate the inhibitory activity of a betaxanthin mixture derived from the transgenic BY-2 line on Aβ aggregation. For betaxanthin mixture, the ThT fluorescence derived from Aβ40 aggregation reduced, indicating that the betaxanthin mixture inhibited Aβ40 aggregation (Figure 4A). Aβ aggregates were also observed by TEM. TEM results were similar to those of the ThT fluorescence assay (Figure 4B, C, D). These findings indicate that Aβ aggregation inhibition is a common bioactivity of betalains.

## Discussion

In this study, we aimed to isolate genes involved in betaxanthin biosynthesis in quinoa and to establish an artificial system for betaxanthin production. Three genes (*CqCYP76AD5v1*, *CqCYP76AD5v2*, and *CqCYP76AD130*), belonging to the β-clade of the *CYP76AD* family, were identified in quinoa, and *CqCYP76AD5v1* and *CqCYP76AD130* were successfully cloned. Agroinfiltration experiments in *Nicotiana benthamiana* revealed that *CqCYP76AD5v1* retains enzymatic activity required for betaxanthin biosynthesis. Furthermore, *CqCYP76AD5v1* and *CqDODA-1* were used to successfully produce betaxanthin on a large scale in tobacco BY-2 cells. The betaxanthin produced was evaluated for its amyloid-β aggregation inhibitory activity and was confirmed to retain this property.

In this study, *CqCYP76AD5v1*, which is involved in betaxanthin synthesis in quinoa, was successfully isolated. This gene is probably involved in betaxanthin biosynthesis in quinoa. Different quinoa varieties produce different pigments (Sandell et al. 2024). In the initial biosynthesis of betalains in quinoa, *CqDODA* is involved in betacyanin and betaxanthin syntheses. On the contrary, *CYP76AD* is responsible for betacyanin synthesis by *CqCYP76AD127*, an α-clade of *CYP76AD*, and betaxanthin by *CqCYP76AD5v1*, a β-clade of *CYP76AD*. This implies that the color differences between quinoa varieties are related to the differential expression of *CqCYP76AD127* and *CqCYP76AD5v1* of the *CYP76AD* family.

In this study, an artificial betaxanthin production system was created by establishing a transgenic line of tobacco BY-2 cells introduced into *CqDODA-1* and *CqCYP76AD5v1*. This betaxanthin-producing line mainly produced vulgaxanthin I, whereas dopaxanthin, miraxanthin V, and indicaxanthin are betaxanthins produced in quinoa grain (Escribano et al. 2017). The discrepancy in betaxanthin composition is attributed to the condensation of betalamic acid with amino acids and other constituents in the betaxanthin biosynthetic pathway via a spontaneous reaction. This is supposed to be contingent upon the amino acid and other substance composition of the host cells responsible for betaxanthin production. The betaxanthin-producing lines established in this study produced 7.1±1.6 µM per liter of tobacco BY-2 cells. In contrast, betaxanthin production in 2-week-old quinoa plants, used to isolate *CqCYP76AD5v1* in this study, was approximately 2.1±0.8 µM per kilogram of hypocotyls (derived from about 23,000 individuals). This suggests that the production of betaxanthin in tobacco BY-2 cells is more efficient than in quinoa. However, previous studies have reported betaxanthin production in tobacco BY-2 cells using genes from plant species other than quinoa (Polturak et al. 2017), with production levels up to 10 times higher than those observed in the present study. These differences in production efficiency can likely be attributed to variations in gene expression methods and the selection of betaxanthin-producing strains. Further improvements in production are expected by optimizing these methods.

Betaxanthin demonstrate anti-inflammatory (Allegra et al. 2014), anti-tumour (Henarejos-Escudero et al. 2020), and anti-aging (Guerrero-Rubio et al. 2020) activities. Previously, we have uncovered the inhibitory effect of betacyanin on Aβ aggregation, a possible cause of Alzheimer’s disease (Imamura et al. 2022). However, the inhibitory effect of betaxanthin on amyloid-β aggregation had not been previously investigated. In the present study, in our search for new biological effects of betaxanthin, we investigated the ability of betaxanthin to inhibit Aβ aggregation. The results showed that a betaxanthin mixture produced in BY-2 cells exerted an inhibitory effect on Aβ aggregation. However, it was not known which betaxanthin molecules inhibited Aβ aggregation, as this result was the activity of a betaxanthin mixture. Betaxanthin was reported to be rapidly transferred into the vasculature (Sawicki et al. 2020). Furthermore, indicaxanthin, one of the betaxanthins, was reported to cross the blood–brain barrier in rats (Allegra et al. 2015). Based on these findings, betaxanthin is expected to play a role in brain function and may have a protective effect against Alzheimer’s disease.

In this study, *CqCYP76AD5v1*, which is involved in betaxanthin biosynthesis in quinoa, was identified and isolated, and betaxanthins were successfully produced in tobacco BY-2 cells. Furthermore, betaxanthin has a novel biological effect by inhibiting Aβ aggregation. However, it was not known which betaxanthin molecules inhibited Aβ aggregation. In conclusion, the present results may open new applications for the natural pigment betaxanthin, such as nutritional supplements.

## Abbreviations

Aβ: amyloid-β
Aβ40: human amyloid beta 1–40 peptide
PCR: polymerase chain reaction
RT: reverse transcription
TEM: transmission electron microscopy
ThT: Thioflavin T

## Description of supplementary files

Supplementary Figure S1. Alignment of the deduced amino acid sequences of the α- and β-clade CYP76AD family.

Supplementary Table S1. Proteins used for phylogenetic analysis.

Supplementary Table S2. List of primers used in this study.
