# Supplementary material for: Identification of CqCYP76AD5v1, a gene involved in betaxanthin biosynthesis in Chenopodium quinoa, and its product, betaxanthin, which inhibits amyloid-β aggregation
Source: Plant Biotechnol (Tokyo). 2025 Jun 25;42(2):111–9. doi: 10.5511/plantbiotechnology.25.0122a (PMC12235425; doi:10.5511/plantbiotechnology.25.0122a)
Supplement: Supplementary Data [file plantbiotechnology-42-2-25.0122a-s001.pdf]

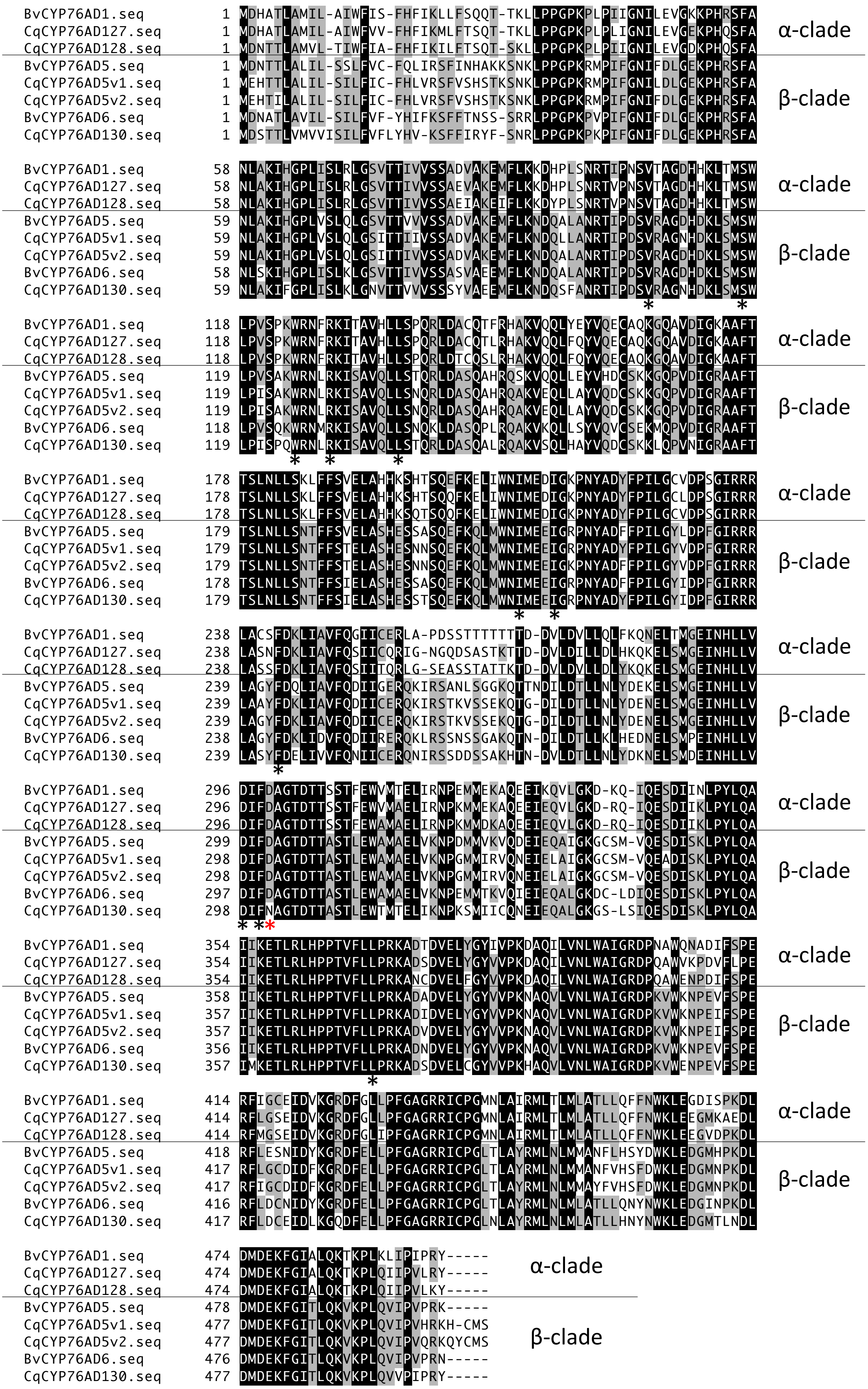

Supplementary Figure S1. Alignment of the deduced amino acid sequences of the α- and β-clade CYP76AD family from quinoa and beet (Table S1). Asterisks indicate amino acid residues expected to form the chemical substrate-binding pocket. The red asterisk highlights amino acids in the chemical substrate-binding pocket that were mutated exclusively in CqCYP76AD130.

Supplementary Table S1. Proteins used for phylogenetic analysis

NCBI Accession No.	Gene Name	Species
XP_021776187	CqCYP76AD5v1	*Chenopodium quinoa*
XP_021717175	CqCYP76AD5v2	*Chenopodium quinoa*
XP_021717192	CqCYP76AD130	*Chenopodium quinoa*
XP_021769302	CqCYP76AD127 (CqCYP76AD1-1)	*Chenopodium quinoa*
XP_021732600	CqCYP76AD128 (CqCYP76AD1-2)	*Chenopodium quinoa*
I3PFJ5	BvCYP76AD1	*Beta vulgaris*
AJD87473	BvCYP76AD5	*Beta vulgaris*
AJD87474	BvCYP76AD6	*Beta vulgaris*
XP_057523307	AtCYP76AD1-like_XP_057523307	*Amaranthus tricolor*
XP_057527403	AtCYP76AD1-like_XP_057527403	*Amaranthus tricolor*
XP_057541335	AtCYP76AD1-like_XP_057541335	*Amaranthus tricolor*
XP_021847068	SoCYP76AD1-like_XP_021847068	*Spinacia oleracea*
XP_021847070	SoCYP76AD1-like_XP_021847070	*Spinacia oleracea*
XP_021848355	SoCYP76AD1-like_XP_021848355	*Spinacia oleracea*
XP_021853416	SoCYP76AD1-like_XP_021853416	*Spinacia oleracea*
XP_021839573	SoCYP76AD1-lke_XP_021839573	*Spinacia oleracea*
XP_021839707	SoCYP76AD1-lke_XP_021839707	*Spinacia oleracea*
AET43292	MjCYP76AD3	*Mirabilis jalapa*
AJD87463	MjCYP76AD5	*Mirabilis jalapa*
AJD87471	MjCYP76AD15	*Mirabilis jalapa*

Supplementary Table S2. Primers used in this study

			Sequence (5´→ 3´)
Cloning	*CqCYP76AD5v1*	Forward	ATGGAACATACAACACTTGCATTGATACTT
		Reverse	TTATGACATGCAGTGTTTCCTATGTACTGG
	CqCYP76AD5v2	Forward	ATGGAACATACAATACTTGCATTGATACTT
		Reverse	TTATGACATGCAATATTGTTTCCTTTGTAC
	CqCYP76AD130	Forward	ATGGATAGTACAACACTTGTTATGGTTGTT
		Reverse	TTAATACCTTGGGATTGGAACAACTTGAAG
Over-expressiong vector	*CqCYP76AD5v1*	Forward	AAAGAATTCATGGAACATACAACACTTGCATTGATACTT
		Reverse	AAAGGATCCTTATGACATGCAGTGTTTCCTATGTACTGG
	CqCYP76AD130	Forward	AAAGAATTCATGGATAGTACAACACTTGTTATGGTTGTT
		Reverse	AAAGGATCCTTAATACCTTGGGATTGGAACAACTTGAAG
RT-PCR	*CqCYP76AD5v1*	Forward	ATGGAACATACAACACTTGCATTGATACTT
		Reverse	TTATGACATGCAGTGTTTCCTATGTACTGG
	CqCYP76AD130	Forward	ATGGATAGTACAACACTTGTTATGGTTGTT
		Reverse	TTAATACCTTGGGATTGGAACAACTTGAAG
	CqDODA1-1	Forward	TGATACTATCTACGATTTTGATGACT
		Reverse	TTCATATGTATTCACTTCTTCAAACC
	AcGFP1	Forward	GGATCCATGGTAAGCAAAGGAGCCGAACTTT
		Reverse	GGTCACCTCACTTATACAGCTCATCCATCCC
	L23	Forward	AGCTGATCCGTCCAAAAAATCTGATCCCA
		Reverse	TTTGTTGGCCACGTCCAACGCATCGTAG
	CesA	Forward	GAAGGTTGGACT ATGCAAGA
		Reverse	ATAGATCCATCCAA TCTCTTTTCCCCA
Sequence	*CqCYP76AD5v1*	Forward	CGTCTTCAAAGCAAGTGGATTG
		Reverse	ATCGCAATGTTTCTTTGATGATGGCTTGCA
		Reverse	TTCCCGATCTAGTAACATAGATGAC
	CqCYP76AD130	Forward	CGTCTTCAAAGCAAGTGGATTG
		Forward	AACGCCTTGATGCTAGCCAAGCCCTAAGGCAAG
		Reverse	TTCCCGATCTAGTAACATAGATGAC
